# Combined System of Activated Sludge and Ozonation for the Treatment of Kraft E_1_ Effluent

**DOI:** 10.3390/ijerph6031145

**Published:** 2009-03-17

**Authors:** Marcia Regina Assalin, Edna dos Santos Almeida, Nelson Durán

**Affiliations:** Laboratório de Química Biológica, Instituto de Química, Universidade Estadual de Campinas CP 6154, CEP 13084-971, Campinas, SP, Brazil; E-Mails: ednasa@cetind.fieb.org.br (E.S.A.); duran@iqm.unicamp.br (N.D.)

**Keywords:** Activated sludge, ozonation, combined processes, Kraft E_1_ effluent

## Abstract

The treatment of paper mill effluent for COD, TOC, total phenols and color removal was investigated using combined activated sludge-ozonation processes and single processes. The combined activated sludge-O_3_/pH 10 treatment was able to remove around 80% of COD, TOC and color from Kraft E_1_ effluent. For the total phenols, the efficiency removal was around 70%. The ozonation post treatment carried out at pH 8.3 also showed better results than the single process. The COD, TOC, color and total phenols removal efficiency obtained were 75.5, 59.1, 77 and 52.3%, respectively. The difference in the concentrations of free radical produced by activated sludge-O_3_/pH 10 and activated sludge-O_3_/pH 8.3 affected mainly the TOC and total phenol removal values.

## 1. Introduction

The rapid increase of population and the increased demand for industrial establishments to meet human needs have created problems such as overexploitation of available resources, leading to pollution of the land, air and water environments [1]. Effluents from chemical plants may contain significant amounts of poorly biodegradable aromatic compounds, which could negatively affect water quality and public health.

The pulp and paper industry is considered to be a serious environmental hazard due to the multiplicity of impurities complexity of the chemical structures present and significant quantities of wastewater generated (about 175 m^3^ of wastewater per ton of paper produced) [1–4]. Among the various sections in pulp and paper mills, the bleaching section is considered to be the most polluted. During this stage, chlorine or chlorine dioxide is used to be bleach pulp and release chlorinated and nonchlorinated compounds from lignin and wood extractives. Typically, these effluents contain high concentrations of chlorophenolic compounds, chloroacetones and chloroform, which are colored and recalcitrant [5,6]. It has been reported that production of one ton of paper contributes 100 Kg of color imparting substances and 2–4 Kg of organochlorines to the bleach plant effluents [2]. The high chemical diversity of these pollutants causes a variety of clastogenic, carcinogenic and mutagenic effects on fishes and other aquatic communities in recipient water bodies [7,8]. Moreover, overall it is a very polluting industry, and efforts must be made to meet current standards related to the quality and quantity of effluent discharge [9–11].

Worldwide, there are many existing biological and chemical treatment processes for paper and board mill effluent such as those based on aerobic, anaerobic, algal, fungal biomass, ozonation, electrochemical, photocatalytsis, coagulation-flocculation treatment, etc. [12–19]. In general, the main treatment process used at pulp and paper mills plants is primary clarification, succeeded in some cases by secondary treatment, generally of a biological nature (anaerobic treatment, aerated lagoons and activated sludge) [20]. These classical treatments have been successful in lowering the chemical and biological oxygen demands (COD, BOD, respectively), but their applicability is limited by a great number of problems. These biologically treated effluents still contain significant amounts of coloured compounds, microorganisms, recalcitrant organics and a minor amount of biodegradable organics, as well as suspended solids. Biological treatment does not significantly reduce the inorganic content in the effluent and desalting is sometimes needed before reuse of the effluents in the manufacturing processes [21]. Given the limitations of the current biological wastewater treatment, there is an increasing interest to develop a more effective treatment approach to reduce the impacts of pulp mill effluents on the environment.

Advanced oxidation processes (AOPs) are among promising technologies that have received increasing interest for the treatment of drinking water and many industrial wastewaters. Among the advanced oxidative processes, the application of ozone in the industrial treatment effluents is also very interesting due to highly oxidizing characteristics. However, in the Kraft effluent treatment the ozonation process results in negligible total organic carbon removal (TOC). At the same time, high ozone doses are necessary, generating appreciable costs [11,12,22–26].

An integrated biological and AOPs processes treatment would provide a viable alternative and could help remove hardly biodegradable recalcitrant organic matter (ROM) from the effluent economically and effectively. Effluents streams containing toxic and inhibitory compounds can be treated by AOPs to produce biodegradable intermediates, which are then readily treated biologically. Beltran *et al.* [22] applied the combined treatment (ozonation coupled with activated sludge) to a diluted sherry stillage, a high strength wastewater. The two ways of coupling ozonation with activated sludge are proposed by authors. Ozonation prior to activated sludge is advised for high-concentration wastewater to reduce polyphenol concentration, thus removing inhibiting effects. For wastewater with low polyphenol concentration the activated sludge-ozonation-activated sludge sequence is preferred to enhance the overall process performance in terms of oxidation efficiency and sludge settling. Chang *et al.* [27] investigated the effects of ozonation on the degradation target in coke–oven wastewater under various pH conditions and the effect on subsequent biological treatment. By observing the BOD_5_/COD ratio (which decreased from 0.52 to about 0.10), it was determined that the pretreatment was not beneficial to the subsequent activated sludge process in terms the biodegradability. The results suggesting that the pre-ozonation could reduce the toxicity impact and benefit the subsequent biological treatment unit. The study realized by Morais *et al.* [28] showed that the combination of ozonation and biological treatment of kraft pulp elemental–free (ECF) bleaching resulted in statistically significant increases in removal of COD, BOD_5_, TOC, Lignin and AOX, compared to the effluent without ozone treatment.

According Biag and Liechti ozonation applied at the end of the industrial treatment line, after biological and physico-chemical stages, achieves an important removal of the residual organic matter in the effluent. The biodegradability enhancement resulting from partial oxidation of paper mill effluents allows an appropriate sequencing of ozonation followed by biofiltrattion, so, the combined oxidation process provides high COD elimination efficiency together with a reduced ozone need [29].

Bijan and Mohseni [30] showed that ozone treatment enhanced the biodegradability of the effluent (from a pulp mill alkaline bleach plant effluent) allowing for a higher removal of pollutants. The conversion of high molecular weight (HMW) to low molecular weight (LMW) compounds was an important factor in the overall biodegradability enhancement of the alkaline effluent.

The objective of this study was to evaluate the efficiency of combining biological (activated sludge) and ozonation(at pH 8.3 and 10) processes for COD, TOC, total phenols and color removal of Kraft E_1_ effluent. The results obtained from single and combined treatments were evaluated and compared.

## 2. Materials and Methods

### 2.1. Paper Mill Effluent

Wastewater was obtained from a bleach Kraft mill located in the Campinas city region (São Paulo State, Brazil). This mill process uses only *Eucalyptus grandis* wood. The sample effluent was obtained after the first alkaline extraction stage (E_1_). The bleaching process utilizes Cl_2_, oxygen and hydrogen-peroxide reinforced alkaline extraction stage, NaClO_4_ and hydrogen peroxide. The samples were taken periodically (approximately once a month) over a three year period. The samples was stored at 4 °C in bottle flasks and used without previous filtration.

### 2.2. Activated Sludge System

An aerobic reactor (2.5 L) was operated as a continuous system for a period of 700 days. The reactor was continuously fed with Kraft E_1_ effluent (at pH 7) by means of a peristaltic pump. The hydraulic retention time (HRT) was maintained in 12 h. The sludge was periodically recycled, and excess sludge which grew during the aeration stage was withdrawn from the bottom to obtain solids retention times (SRT) in 10 days. Inside the reactor, the oxygen concentration was maintained above 5 mg L^–1^ by an aquarium-type air pump with sintered-sand diffusers at the bottom of the reactor. The adequate nutrient balance is necessary to obtain sufficient biodegradation. An estimate of requirements for N and P is based on the fact that wasted mixed liquor volatile suspended solids (MLVSS) contains approximately 12% of its dry weight, as N and 2% as P [39]. N as urea and P as KH_2_PO_4_ were supplied to maintain a C:N:P ratio of 100:5:1. Dissolved oxygen, pH, temperature, total suspended solids and volatile suspended solids were regularly monitored and adjusted when necessary [31,32].

### 2.3. Ozonation Process

Ozone was generated from pure oxygen using an OZOCAV ZT-2 generator (Inter Ozone Ingeneria Ecologica, Santiago, Chile). The amount of ozone produced was determined spectrophotometrically at 258 nm (ɛ =3.000 L mol^–1^cm^–1^) in the gas phase by passing the mixture of oxygen and ozone through a flow cell. The system reached a steady-state production of ozone in 10 min [33,34]. An ozone concentration of 14 mg L^–1^ was applied for 1 hour in a batch reactor. Lab-scale investigations were done with paper mill wastewater. Samples of this effluent (400 mL) were submitted to the ozonation process (at pH from activated sludge 8.3 and adjusted to pH 10) at room temperature, using a tubular 500 mL reactor fitted with a sintered glass dispenser that released the gas from the bottom of the reactor. Ozonation was continued until the consumption of ozone had ceased. For subsequent experiments, an excess of ozone gas was passed from the reactor into a glass flask containing a 2% solution of KI.

### 2.4. Analytical Control

All samples were submitted to color, total phenols, chemical oxygen demand (COD) and total organic carbon (TOC) analyses. The effluent discoloration was evaluated by measuring the absorbance at 465 nm at pH 7.6 with a Hitachi U-200 spectrophotometer [3]. Total phenol concentrations (determined by the Folin-Ciocalteu reaction), SSV, COD, N and P were determined according to Standard Methods (APHA) [35] TOC was determined using Shimadzu TOC-5000A apparatus. Before measurements, all the samples were filtered with Millipore Millex nonsterile syringe filters which had 0.45 μm pore size PVDF membrane.

## 3. Results and Discussion

In this work, Kraft effluent treatment was evaluated by combining in sequence activated sludge and ozone processes. The Kraft effluent samples were first submitted to biological treatment and then ozonized at pH 8.3 and 10 for 60 minutes. The physical-chemical parameters (TOC, COD, total phenols and color) were measured during both processes. The characteristics of the Kraft E_1_ effluent used in this study are described in Table 1.

Prior to this experiment in a continuous activated sludge reactor, the biomass from domestic wastewater was acclimated to the substrate of interest (Kraft effluent), for 39 days to permit the growth of the microorganisms which can use the Kraft E_1_ effluent as a carbon source. Acclimation is widely used to maximize the removal efficiency of specific compounds that are difficult to biodegrade. The steady-state condition was verified when the average of the consecutive measurements for COD parameter showed a standard deviation less than 5%.

The effect of aeration time on the COD removal was studied under three different periods of aeration (20, 12 and 6), achieving between 47.7 and 70% COD removal when HRT was varied from 6 to 20 h. Phenolic compounds inhibit the biomass present in biological treatment systems and to degrade them it is necessary to maintain a higher HRT than for organic matter coming from COD [32]. Despite the benefits of the longer HRT, it is unpractical to extend the aeration time endlessly due to the increase of operation costs [4]. For this study, the HRT was maintained in 12 h.

The biomass concentration has an important effect on treatment performance. Previous studies showed that a minimum of mixed liquor suspended solids (MLSS) in the activated sludge process for of paper mill effluent treatment is 2,000–2,500 mg L^–1^ [1]. Tsang *et al.* [4] showed that a higher concentration of MLSS promoted the activated sludge performance in organic parameters removals, however the improvement of COD removal efficiency from increasing MLSS was limited within a certain range. The mixed liquor suspended solids (MLSS) in this study, was 5,300 – 6,200 mg L^–1^ %. Some negative effects on the effluent quality, such as excess air supply for aeration, long sludge settling time and high concentration of suspended solids in the effluent can occur due to high biomass concentration in the reactor [4] For this range of COD removal the F/M ratio (food /microorganism) varied from 0.21 to 0.80 g COD/gVSS d (volatile suspended solids). According to the literature for activated sludge systems with conventional aeration, for treatment of paper mill effluent, the optimal F/M ratios falls beween 0.09 and 0.98 g COD/gSSV d [36].

For total phenolic compounds the removal achieved with the biological process was between 12–15%. It is well known that phenolic compounds inhibit the biomass present in biological treatment systems. Therefore, to degrade them, it is necessary to maintain a higher HRT and use acclimated biomass.

Color removal efficiency by the biological process was low (less than 12%). Color results from the presence of the lignin or polymerized tannins, compounds with high molecular weight, which are non- toxic but poorly biodegradable. Typically, an activated sludge system is composed of bacteria flocs that produce intracellular enzymes, responsible for biodegradation. This is the reason for the low biodegradability ratio for compounds with high molecular weight [31,37].

The effluent obtained after biological treatment was submitted to the ozonation process at different pH values (8.3 and 10) for 60 minutes. The ability of the hydroxyl radical to break down the molecular structure of the chemical compounds is advantageous for partial oxidation of non-biodegradable high molecular weight substances [38].

**Figure 1.**
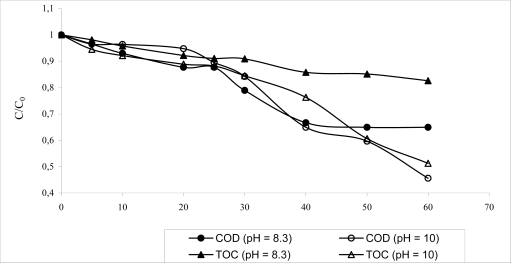
COD and TOC removal by ozonation process (at pH 8.3 and 10) after biological treatment.

**Figure 2.**
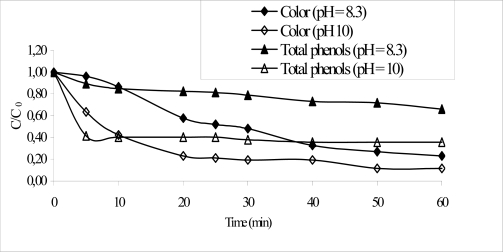
Color and Total phenols removal by the ozonation process (pH 8.3 and 10) after biological treatment.

The Figure 1 shows the COD and TOC results obtained for the combined system (activated sludge-O_3_/pH 8 and activated sludge-O_3_/pH 10). The O_3_/pH 10 ozonation process was more efficient, achieving 54.4 and 48.7% for COD and TOC removal, respectively. The O_3_/pH 8.3, showed only 35.1 and 17.4% efficiency removal for these parameters. This indicates that the ozonation process at pH 10 was able to remove remaining refractory fraction, increasing the COD and TOC ratio removal. For the removal of phenolic compounds the pH of the ozonation process seriously affected the efficiency of the process. For the systems O_3_/pH 8.3 and O_3_/pH 10, the efficiency removal was 48.5 and 74%, respectively after 60 minutes. At pH 10 deprotonation of phenol (pKa = 10) occurs and the ozone rate constant increases by as much as six orders of magnitude [5]. The main advantage of the application of ozone in the treatment of paper effluent was the color removal, with 76.9 and 88.5% reduction after 60 minutes for ozonation carried out at pH 8.3 and 10, respectively. Figure 2 shows the efficiency removal for both parameters. It is possible observed that the treatment time can be reduced for 20 minutes without seriously affecting the efficiency of the ozonation process. It is an important result, since this implies costs reductions.

The pH of effluent after ozonation treatment was observed to decrease significantly in the process carried out at pH 10 (end pH 8.3). This can be attributed to oxalic acid formation during the ozonation process which explains the reason that this treatment cannot achieve total mineralization after a biological pre-treatment.

The activated sludge/O_3_/pH 8.3 was less efficient than the system carried out at pH 10. After the removal of biodegradable compounds by activated sludge process, there is less competition among compounds present in the treated effluent for hydroxyl radicals formed by ozone decomposition [10,24].

The only difference between the combined processes is the hydroxyl ions concentrations present to initiate–free radical production. Therefore, if the medium is more alkaline, more free radicals are formed and process is more efficient [10].

**Figure 3.**
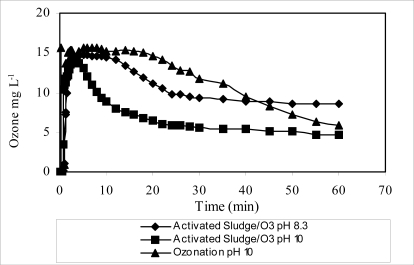
The ozone consumed during the combined processes (with ozonation at pH 8.3 and pH 10) and single ozonation process (at pH 10).

In addition, from an economical point of view, the activated sludge-ozonation/pH 10 system was the most efficient process tested, due to the lower ozone consumption. The ozone consumption decreased from 612.40 mg to 391.8 mg, respectively, for the ozonation carried out at pH 8.3 and 10 after 60 minutes of treatment. The treatment can be interrupted after 20 minutes, when ozone no longer reacts significantly with paper mill effluent species. Besides this, the ozone consumption by the single ozonation process (673.2 mg after 60 minutes of treatment) is larger than any of the combined processes studied as shown in Figure 3. This indicates that the ozone was consumed in combined processes only by the refractory compounds.

## 4. Conclusions

An activated sludge/O_3_/pH 10 treatment was able to remove over 80% of COD, TOC and color from Kraft E_1_ effluent. Considering only the total phenols, the efficiency removal was around 70%. The ozonation post-treatment carried out at pH 8.3 also showed better results than the single process. The COD, TOC, color and total phenols removal efficiencies obtained were 75.5, 59.1, 77 and 52.3%, respectively.

From the comparison of the results obtained by combined processes and single processes, it is possible to conclude that only the use of combined processes can be applied to increase the degradation ratio for all the studied parameters, with less ozone consumption and consequently, cost reduction.

## Figures and Tables

**Table 1.** Initial Characteristics of the alkaline bleach plant effluent.

COD (mg L^–1^)	1,500–2,500
TOC (mg L^–1^)	500–1,000
Color (at 465 nm)	0.15–0.30
pH	9.8–10.2

**Total phenol (mg L^−1^)**	10–30
